# AngioMatrix, a signature of the tumor angiogenic switch-specific matrisome, correlates with poor prognosis for glioma and colorectal cancer patients

**DOI:** 10.18632/oncotarget.2470

**Published:** 2014-10-07

**Authors:** Benoit Langlois, Falk Saupe, Tristan Rupp, Christiane Arnold, Michaël van der Heyden, Gertraud Orend, Thomas Hussenet

**Affiliations:** ^1^ Inserm U1109, MN3T team, Molière, Strasbourg, 67200, France; ^2^ Université de Strasbourg, Strasbourg, 67000, France; ^3^ LabEx Medalis, Université de Strasbourg, Strasbourg, 67000, France; ^4^ Fédération de Médecine Translationnelle de Strasbourg (FMTS), Strasbourg, 67000, France

**Keywords:** Tumor angiogenesis, angiogenic switch, extracellular matrix, matrisome

## Abstract

Angiogenesis represents a rate-limiting step during tumor progression. Targeting angiogenesis is already applied in cancer treatment, yet limits of anti-angiogenic therapies have emerged, notably because tumors adapt and recur after treatment. Therefore, there is a strong need to better understand the molecular and cellular mechanisms underlying tumor angiogenesis. Using the RIP1-Tag2 transgenic murine model, we identified 298 genes that are deregulated during the angiogenic switch, revealing an ingression/expansion of specific stromal cell types including endothelial cells and pericytes, but also macrophages and perivascular mesenchymal cells. Canonical TGF-β signaling is up-regulated during the angiogenic switch, especially in tumor-associated macrophages and fibroblasts. The matrisome, comprising extracellular matrix (ECM) and ECM-associated molecules, is significantly enriched, which allowed us to define the AngioMatrix signature as the 110 matrisomal genes induced during the RIP1-Tag2 angiogenic switch. Several AngioMatrix molecules were validated at expression level. Ablation of tenascin-C, one of the most highly induced ECM molecules during the switch, resulted in reduced angiogenesis confirming its important role. In human glioma and colorectal samples, the AngioMatrix signature correlates with the expression of endothelial cell markers, is increased with tumor progression and finally correlates with poor prognosis demonstrating its diagnostic and therapeutic potential.

## INTRODUCTION

Angiogenesis, a fundamental biological process by which novel blood vessels are formed from pre-existing ones [1], represents a rate-limiting step during tumor progression [2]. Studies from murine models have indicated that angiogenesis occurs relatively early along tumor formation and progression [2]. In particular, the murine RIP1-Tag2 model of pancreatic neuroendocrine tumorigenesis (PNET; ref. [3]) has recurrently allowed to gain novel insights into the molecular and cellular mechanisms governing tumor angiogenesis and progression. In this model of multistep tumorigenesis, pancreatic beta cells of the Langerhans islets over-express the SV40 T antigen oncogene which stochastically drives the sequential transformation of a fraction of normal islets into hyperplastic, angiogenic and macroscopic tumor islets [2]. This *in vivo* PNET model was key to provide evidences demonstrating that a fraction of islets undergoes an angiogenic switch early during tumor progression [4]. Several molecular and cellular mechanisms were described to promote the RIP1-Tag2 angiogenic switch. These include the crucial role of VEGFA and its signaling [5] and in particular, the matrix metalloprotease MMP-9-mediated regulation of VEGF bioavailability [6]. Neutrophils appear to be a source of MMP-9 hereby promoting the angiogenic switch [7, 8].

The RIP1-Tag2 model is widely used in a pre-clinical setting to evaluate anti-tumor therapeutic strategies, including angiogenesis inhibitors [9–15]. Importantly, several key conceptual advances in our understanding of how tumors adapt and become resistant to anti-angiogenic therapies, a major clinical challenge that has emerged [16, 17], were also obtained using this model [18, 19].

Here we used a genome-wide gene expression profiling strategy to uncover potential novel mechanisms underlying the angiogenic switch during RIP1-Tag2 tumor progression. We show that the angiogenic switch is associated with the deregulation of a limited number of genes, some of which reflect the expansion and ingression of stromal cells and the activation of canonical TGF-β signaling in tumor-associated macrophages and fibroblasts. Furthermore, a significant part of these genes encodes ECM and ECM-associated molecules, together defining the AngioMatrix signature. We show that this signature correlates with endothelial cell (EC) markers and tumor progression in human colorectal cancer (CRC) and glioma. Finally, its high expression correlates with poor prognosis for CRC, low grade glioma and glioblastoma (GBM) patients.

## RESULTS

### Gene expression profiling of the tumor angiogenic switch in a murine PNET model

**Figure 1:**
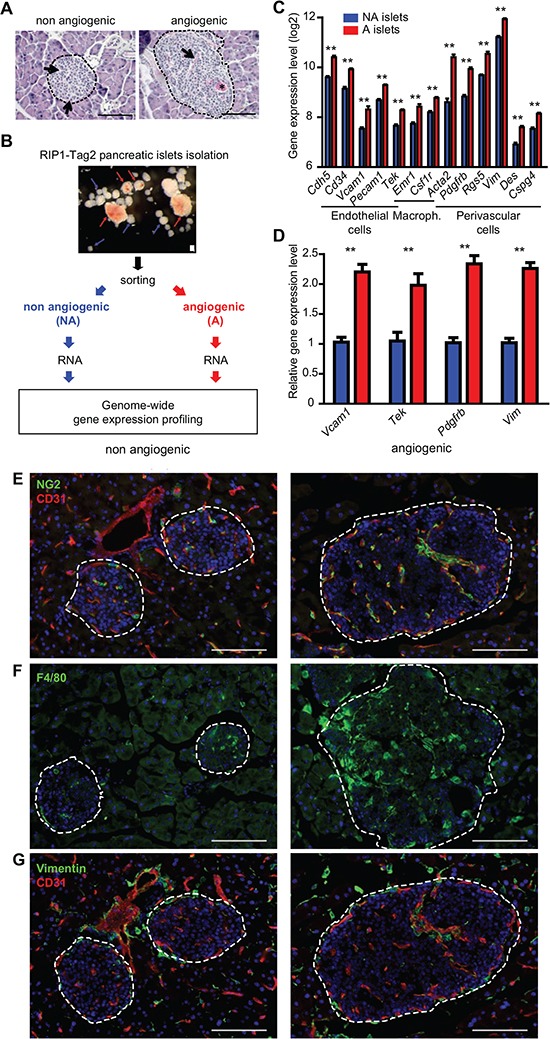
Transcriptomic profiling of the RIP1-Tag2 angiogenic switch reveals the up-regulation of stromal cell markers **(A)** patterns of non-angiogenic (left) and angiogenic (right) islets in H&E stained tissue sections from RIP1-Tag2 pancreata. Examples of normal capillaries in a non angiogenic islet (arrows) and of a dilated vessel (arrow) and micro-hemorrhaging (asterisk) in the angiogenic islet. **(B)** strategy used to compare angiogenic and non angiogenic pancreatic tumor islets by gene expression profiling upon their differential isolation, sorting and RNA extraction. **(C)** up-regulation of specific stromal cell markers in the transcriptome of RIP1-Tag2 angiogenic (red) compared to non angiogenic (blue) islets: markers for EC (*Cdh5*, *Cd34*, *Vcam1*, *Pecam1*, *Tek* or Tie2, also expressed by macrophages), macrophages/monocytes (*Emr1*, *Tek* and *Csf1r*) perivascular and smooth muscle cells (*Acta2*, *Pdgfrb*, *Rgs5*, *Vim*, *Des* and *Cspg4* encoding NG2). Measures represent the mean expression level from two independent profiling experiments, error bars the SEM. ** p < 5×10^−3^. **(D)** RT-qPCR confirmation of the up-regulation of stromal cell markers (*Vcam1*, EC; *Tek*: ECs and macrophages/monocytes; *Pdgfrb*: pericytes; *Vim*: perivascular SMC) in angiogenic (red) compared to non angiogenic (blue) islets. Measures represent the mean expression level from two independent experiments, error bars the SEM. ** p < 5×10^−3^. **(E-G)** expression (immunofluorescence) of stromal cell markers in non angiogenic and angiogenic islets, CD31 and NG2 (E), F4/80 (F) and Vimentin and CD31 (G). Nuclei are stained with Dapi (blue). In A and E-G dashed lines encircle islets. Scale bars, 100 μm.

We used the RIP1-Tag2 mouse model as a prototypical *in vivo* model of the tumor angiogenic switch [2, 20] to comprehensively address the underlying mechanisms. We chose an early time point (8 weeks) when a subset of pancreatic islets has undergone the angiogenic switch (Fig. 1A and Supplementary Fig. S1A) but had not yet progressed into macroscopic tumors. Islets were isolated from RIP1-Tag2 mice and classified as angiogenic or non angiogenic based on their appearance. RNA was extracted from the isolated islets to determine genome-wide gene expression levels using microarrays (Fig. 1B). The comparison of the transcriptome of non angiogenic *versus* angiogenic islets yielded a restricted list of 298 significantly deregulated genes, the “AngioSwitch signature”. We first noted that this signature included several markers of stromal cells (Fig. 1C). Characteristic markers of EC (e.g. *Pecam1* and *Cdh5* encoding CD31 and VE-cadherin, respectively), perivascular cells (*Acta2* encoding alpha-smooth muscle actin or αSMA, *Cspg4* encoding NG2, *Pdgfrb*) and monocytes/macrophages (*Emr1* encoding F4/80, *Csf1r*) were found up-regulated in angiogenic islets, which was confirmed by RT-qPCR (Fig. 1D) and tissue staining (Fig. 1, E-G and Supplementary Fig.S1, B-D).

### Stromal-specific activation of canonical TGF-β signaling during the RIP1-Tag2 angiogenic switch

**Figure 2:**
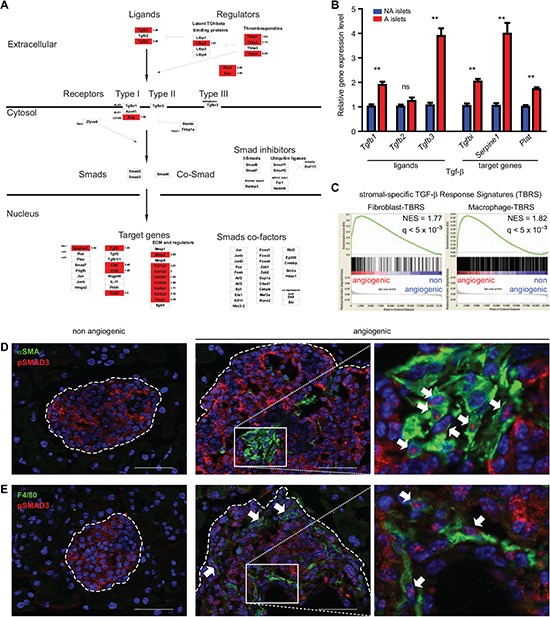
TGF-β signaling activation during the RIP1-Tag2 angiogenic switch **(A)** schematic depiction of gene expression profiling data on a TGF-β signaling GenMapp. Genes (represented by boxes) in red indicate genes significantly up-regulated in angiogenic islets. No gene in this pathway was down-regulated. Several TGF-β ligands, extracellular regulators, the *Endoglin* co-receptor (*Eng*, encoding CD105), together with target genes are up-regulated in angiogenic islets. **(B)** RT-qPCR analysis of TGF-β ligands and target gene expression in non angiogenic (blue) and angiogenic (red) islets. The *Tgfb1* and *Tgfb3* genes are significantly up-regulated in angiogenic islets together with the prototypical Smad2/3 target genes *Tgfbi*, *Serpine1* and *Plat*. Measures represent the mean of two independent experiments, error bars the SEM, ** p < 5×10^−3^, ns not significant. **(C)** GSEA demonstrates significant enrichment of fibroblast- and macrophage- specific TGF-β response signatures in the transcriptome of angiogenic islets. The Normalized Enrichment Score (NES) and the FDR q-value assessing the significance of enrichment are indicated. **(D-E)** co-staining of phosphorylated SMAD3 (pSMAD3) with αSMA (D) or F4/80 (E) in RIP1-Tag2 islets. Nuclear localization of pSMAD3 is observed in angiogenic islets, predominantly in tumor-associated αSMA+ fibroblasts (D) and F4/80+ macrophages (E) (arrows). Nuclei are stained in blue (DAPI). Dashed lines encircle islets; non angiogenic: left column, angiogenic: middle column and higher magnification pictures corresponding to the boxed areas within angiogenic islets are presented (right column). Scale bars, 50 μm.

Several TGF-β pathway members and target genes were found up-regulated, including ligands and extracellular regulators, *Cd105* (encoding endoglin, a TGF-β co-receptor) and known target genes (Fig. 2A). The up-regulation of genes encoding TGF-β ligands (*Tgfb1* and *Tgfb3*) and prototypical SMAD2/3 target genes (*Tgfbi*, *Serpine1* and *Plat* encoding PAI-1 and t-PA, respectively) was confirmed by RT-qPCR (Fig. 2B). We hypothesized that TGF-β signaling may occur preferentially within stromal cells, as a previous study revealed the presence of ALK5 (Tgf-β receptor 1)-positive cells of presumably stromal origin within RIP1-Tag2 angiogenic islets [21], which suggested that these unidentified stromal cell type(s) could undergo canonical TGF-β signaling. We used Gene Set Enrichment Analysis (GSEA) to determine the enrichment of stromal cell-specific TGF-β response signatures (TBRS; ref [22]) and found that the fibroblast- and the macrophage-specific TBRS were significantly enriched in angiogenic islets (Fig. 2C), suggesting that these stromal cell types may undergo canonical TGF-β signaling. To test this hypothesis, we analyzed the expression and sub-cellular localization of SMAD3 phosphorylated on S423/S425 (pSMAD3), as readout for TGF-β signaling activation, in RIP1-Tag2 tissue sections co-stained with stromal markers. While in non angiogenic islets an exclusively cytoplasmic staining was observed in some cells, within angiogenic islets pSMAD3 expression and nuclear localization was recurrently detected in some tumor cells but also more strikingly in both αSMA-positive fibroblasts and F4/80-positive macrophages (Fig. 2, D and E), demonstrating that these stromal cells undergo canonical TGF-β signaling in the angiogenic islets.

### Up-regulation of ECM and ECM-associated genes during the RIP1-Tag2 angiogenic switch: identification of the AngioMatrix signature

**Figure 3:**
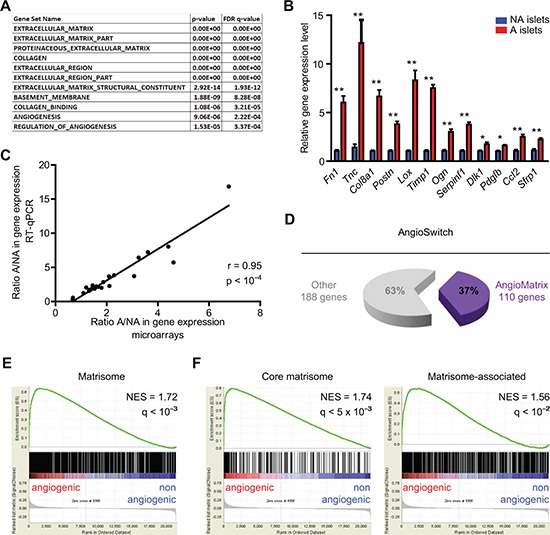
Genes encoding the extracellular matrix and regulators, or matrisome, are up-regulated during the RIP1-Tag2 angiogenic switch **(A)** significantly enriched GO categories in the AngioSwitch signature. The p- and FDR q-values indicate the significance of enrichment. **(B)** RT-qPCR validation of increased expression for 12 candidate genes up-regulated in angiogenic islets. Data (blue, non-angiogenic; red, angiogenic islets) represent mean and error bars the SEM from two independent experiments. **, p < 5×10^−3^; *, p < 10^−2^. **(C)** comparison of the gene expression ratio determined by array profiling and RT-qPCR for 25 validated genes (22 up-regulated, 1 unchanged and 2 down-regulated). The Pearson correlation coefficient and the p-value are indicated. **(D)** overlap between the AngioSwitch signature and the matrisome: 37% of genes induced during the angiogenic switch belong to the matrisome, defining the AngioMatrix signature (110 genes). **(E-F)** GSEA demonstrate significant enrichment of the matrisome (E) and its divisions (F) in the transcriptome of angiogenic islets. The Normalized Enrichment Score (NES) and the FDR q-value assessing the significance of enrichment are indicated.

We then addressed whether groups of functionally-related genes are over-represented in the AngioSwitch signature using GeneOntologies (GO). This revealed significant enrichment of angiogenesis-related GO categories, supporting the biological relevance of the profiling data, but also of several GO categories related to ECM and secreted molecules (Fig. 3A). By RT-qPCR we validated the up-regulation of 12 of these genes (Fig. 3B), leading to a total of 25 validated genes with a significant correlation between array and RT-qPCR data (Fig. 3C).

**Figure 4:**
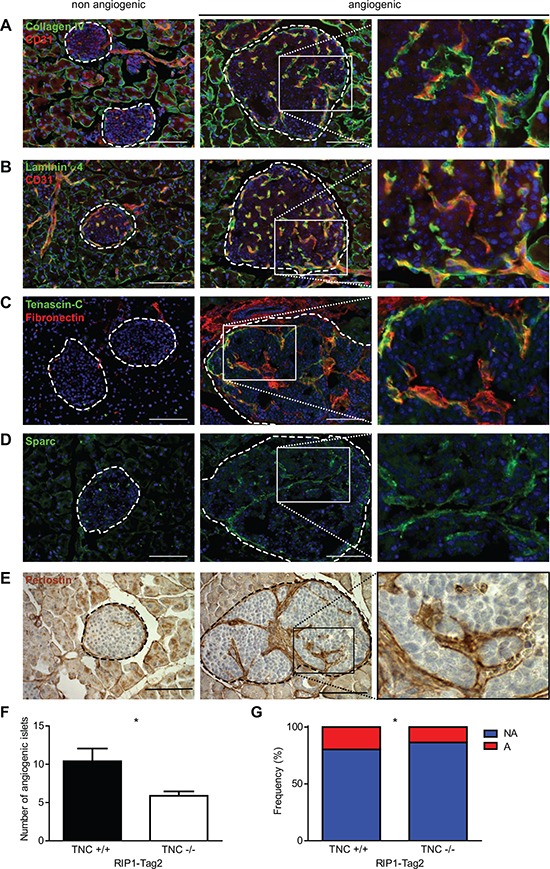
Analysis of AngioMatrix protein expression and functional validation of tenascin-C role in the RIP1-Tag2 angiogenic switch **(A-E)** expression pattern of the vascular basement membrane components collagen IV (A) and laminin α4 (B), and of the ECM glycoproteins fibronectin and tenascin-C (C), sparc (D) and periostin (E). Dashed lines encircle islets; non angiogenic: left column, angiogenic: middle column and higher magnification pictures corresponding to the boxed areas within angiogenic islets are presented (right column). Scale bars, 100 μm. **(F-G)** functional validation of tenascin-C contribution to the angiogenic switch. The number (F) and relative proportion (G) of angiogenic islets are significantly decreased in RIP1-Tag2 TNC −/− mice (n = 8 mice) as compared to TNC +/+ controls (n = 5 mice). A: angiogenic islets, NA: non angiogenic islets. * p < 5×10^−2^.

As GO analysis revealed enrichment of several ECM-related categories, we examined the overlap of the AngioSwitch signature with the matrisome [23, 24], a comprehensive list of genes coding for ECM molecules and regulators. Of note, 37% of the genes composing the AngioSwitch signature encode matrisomal proteins (Fig. 3D), and core matrisomal genes are particularly over-represented (Supplementary Fig. S2). Moreover, GSEA revealed significant enrichment of the whole matrisome and its subclasses the core matrisome and matrisome-associated divisions (Fig. 3, E and F). We further defined the AngioMatrix signature as the 110 matrisomal genes induced during the RIP1-Tag2 angiogenic switch (Table 1). The expression of several AngioMatrix molecules, including vascular basement membrane components (collagen IV, laminin α4) and ECM glycoproteins (fibronectin, periostin, tenascin-C and sparc), was confirmed by tissue staining, which revealed their strong and stromal perivascular expression in angiogenic islets (Fig. 4 and Supplementary Fig. S3). Furthermore, we generated RIP1-Tag2 mice knocked-out for tenascin-C (TNC; ref. [25]), an ECM glycoprotein that was among the most highly up-regulated AngioMatrix genes (Fig. 3B and Table 1). We compared the number of angiogenic islets and the relative proportion of non-angiogenic and angiogenic islets from control and TNC-depleted RIP1-Tag2 mice on tissue sections, which revealed a significant decrease in the absence of TNC (Fig. 4, F and G).

**Table 1:** Composition of the AngioMatrix signature Gene symbol, expression ratio during the RIP1-Tag2 angiogenic switch and matrisome classification are indicated. Genes are grouped by matrisome division and categories, and ranked in descending order of expression ratio.

			Matrisome	
Gene symbol	Ratio A / NA	p-value	Division	Category
**Col8a1**	**3.25**	**1.1869E-07**	**Core matrisome**	**Collagens**
**Col10a1**	**3.11**	**9.3207E-04**		
**Col1a1**	**2.95**	**1.5235E-08**		
**Col1a2**	**2.77**	**1.0141E-09**		
**Col6a3**	**2.26**	**5.9958E-08**		
**Col12a1**	**2.22**	**4.7032E-08**		
**Col14a1**	**2.01**	**5.4409E-06**		
**Col3a1**	**1.91**	**1.4790E-07**		
**Col15a1**	**1.87**	**6.7506E-08**		
**Col4a2**	**1.74**	**4.5137E-06**		
**Col6a1**	**1.71**	**8.8071E-07**		
**Col5a2**	**1.68**	**6.1916E-07**		
**Col6a2**	**1.67**	**3.2904E-06**		
**Col4a1**	**1.57**	**5.2897E-07**		
**Col18a1**	**1.40**	**1.9590E-03**		
**Thbs4**	**6.77**	**3.9919E-09**		**ECM Glycoproteins**
**Fn1**	**4.61**	**6.5803E-08**		
**Tnc**	**3.71**	**1.2366E-08**		
**Postn**	**3.07**	**7.7478E-08**		
**Mfap5**	**2.87**	**3.2983E-09**		
**Fbn1**	**2.74**	**7.9111E-08**		
**Ctgf**	**2.34**	**7.4667E-07**		
**Srpx2**	**2.28**	**7.0478E-07**		
**Cilp**	**2.22**	**3.0748E-06**		
**Svep1**	**2.21**	**1.0975E-07**		
**Mgp**	**2.21**	**3.7883E-05**		
**Thbs2**	**2.12**	**8.1086E-07**		
**Spon1**	**2.04**	**5.5033E-06**		
**Nid1**	**1.98**	**1.4637E-07**		
**Ltbp2**	**1.95**	**1.9311E-05**		
**Pcolce**	**1.92**	**1.9741E-08**		
**Mfap4**	**1.91**	**2.4071E-07**		
**Thbs1**	**1.90**	**3.1110E-04**		
**Lama4**	**1.86**	**4.4791E-07**		
**Sparc**	**1.83**	**1.0330E-07**		
**Aebp1**	**1.71**	**1.4840E-05**		
**Lama2**	**1.71**	**8.5452E-06**		
**Dpt**	**1.70**	**1.5983E-03**		
**Gas6**	**1.69**	**1.7581E-04**		
**Sparcl1**	**1.64**	**1.3306E-04**		
**Slit2**	**1.63**	**2.5898E-07**		
**Lamc1**	**1.58**	**9.9984E-08**		
**Eln**	**1.58**	**8.1302E-05**		
**Igfbp4**	**1.57**	**5.5755E-04**		
**Fbln5**	**1.56**	**1.5923E-04**		
**Fbln2**	**1.49**	**1.3614E-06**		
**Tgfbi**	**1.48**	**2.0248E-05**		
**Mmrn2**	**1.48**	**3.4566E-05**		
**Wisp1**	**1.46**	**1.5947E-03**		
**Igfbp5**	**1.44**	**3.2103E-03**		
**Nid2**	**1.44**	**1.3026E-05**		
**Slit3**	**1.43**	**4.6296E-05**		
**Pxdn**	**1.41**	**8.0373E-06**		
**Lum**	**2.60**	**2.9885E-05**		**Proteoglycans**
**Fmod**	**2.53**	**3.8582E-05**		
**Bgn**	**2.36**	**5.6766E-08**		
**Aspn**	**1.91**	**1.0662E-06**		
**Ogn**	**1.87**	**1.0220E-02**		
**Prelp**	**1.77**	**2.5035E-04**		
**Dcn**	**1.66**	**1.9182E-02**		
**Vcan**	**1.62**	**7.8882E-05**		
**Hspg2**	**1.45**	**1.0067E-04**		
**Lox**	**4.41**	**1.0907E-07**	**Matrisome-associated**	**ECM Regulators**
**Timp1**	**3.61**	**1.0843E-06**		
**Mmp2**	**2.70**	**6.4945E-05**		
**Serpine1**	**2.29**	**8.7933E-07**		
**Adamts2**	**2.17**	**9.2591E-07**		
**Serpinf1**	**2.10**	**1.9858E-06**		
**Serpine2**	**2.03**	**2.6508E-05**		
**Loxl1**	**1.97**	**3.9437E-07**		
**Mmp14**	**1.94**	**9.5446E-07**		
**Ctsc**	**1.70**	**1.5301E-05**		
**Ctsh**	**1.69**	**3.0313E-02**		
**Serpinh1**	**1.65**	**1.5995E-05**		
**Adamts12**	**1.61**	**2.0488E-06**		
**Adamts5**	**1.59**	**1.0358E-05**		
**Adam12**	**1.57**	**4.4749E-07**		
**Adamts1**	**1.52**	**5.9751E-06**		
**Adamtsl3**	**1.49**	**3.8709E-05**		
**Mmp13**	**1.48**	**4.2445E-04**		
**Cd109**	**1.47**	**1.1918E-04**		
**Serpina3n**	**1.47**	**6.5008E-03**		
**Sulf2**	**1.43**	**1.1640E-04**		
**Loxl3**	**1.40**	**3.8243E-05**		
**Adamts9**	**1.40**	**1.9618E-04**		
**Anxa1**	**2.20**	**1.9866E-05**		**ECM-affiliated Proteins**
**Clec4n**	**2.11**	**7.5384E-04**		
**Plxdc2**	**1.81**	**5.2764E-08**		
**Frem1**	**1.78**	**4.3323E-06**		
**Anxa3**	**1.77**	**4.4351E-03**		
**Lgals1**	**1.73**	**1.0923E-06**		
**Plxnd1**	**1.73**	**1.2998E-04**		
**Anxa2**	**1.72**	**2.6193E-05**		
**Colec12**	**1.57**	**6.4004E-04**		
**Cspg4**	**1.48**	**3.5828E-05**		
**Clec7a**	**1.45**	**9.1353E-05**		
**C1qb**	**1.42**	**2.8805E-04**		
**Sema6a**	**1.41**	**3.1453E-06**		
**Gpc6**	**1.41**	**9.0537E-07**		
**Fstl1**	**2.92**	**1.0280E-05**		**Secreted Factors**
**Tgfb3**	**2.09**	**5.2714E-07**		
**Igf1**	**1.93**	**5.8883E-07**		
**Sfrp1**	**1.80**	**4.0155E-03**		
**Cxcl9**	**1.63**	**8.7549E-03**		
**Ccl3**	**1.57**	**2.1757E-03**		
**S100a6**	**1.54**	**1.7308E-04**		
**Angptl2**	**1.45**	**1.2141E-04**		
**Ccl2**	**1.45**	**1.7686E-04**		
**Pdgfc**	**1.42**	**9.5455E-04**		
**Tgfb1**	**1.42**	**1.8128E-03**		

### Expression of the AngioMatrix signature correlates with angiogenesis markers, tumor progression and poor prognosis for CRC, low grade glioma and GBM patients

To address the potential relevance of the AngioMatrix signature for cancer patients, we analyzed transcriptomic datasets, as this strategy enables investigating large and independent patient cohorts. Since insulinoma is rare and mostly benign (and no dataset could be retrieved), we focused on colorectal cancer and glioma, as their incidence is higher, angiogenesis is known to drive their progression and several independent datasets could be retrieved for CRC [26–30] and glioma [31–33].

**Figure 5:**
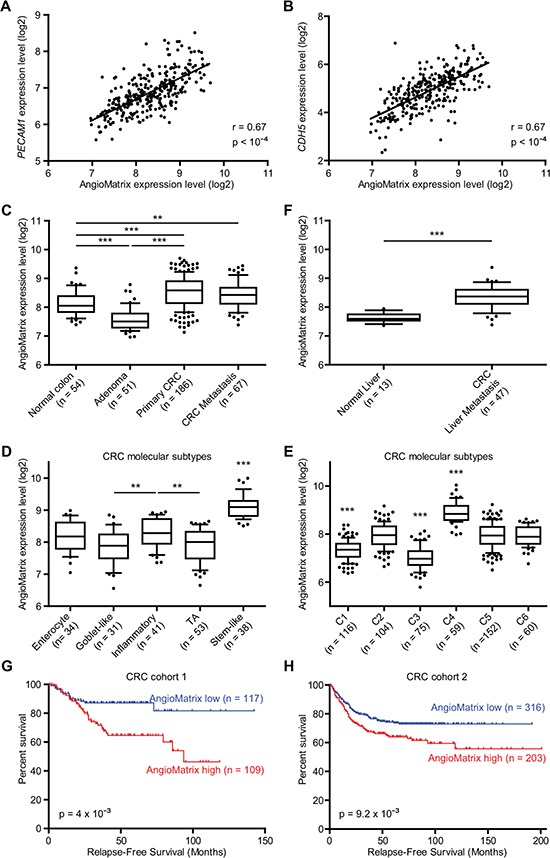
Correlation between AngioMatrix signature expression and EC markers, tumor progression and poor prognosis in human CRC **(A-B)** correlation between AngioMatrix expression level and *PECAM1* (A) or *CDH5* (B) expression in normal, adenoma and primary CRC samples. The Pearson correlation coefficient and the p-value are indicated. **(C, F)** analysis of AngioMatrix expression during CRC progression. Comparison of normal colon, adenoma, primary CRC and CRC metastasis (C) and CRC metastasis *versus* normal liver (F). **(D-E)** analysis of AngioMatrix expression in the different primary CRC molecular subytpes. Note the significant higher levels of AngioMatrix in the Stem-like (D) and C4 (E) subtypes. In C-F, ***and ** indicate p-values < 10^−3^ and 10^−2^, respectively. **(G-H)** Kaplan-Meier survival analysis of CRC patients. Patients were stratified according to the average expression of the AngioMatrix signature as AngioMatrix high or low using a cutoff value. In each cohort, high AngioMatrix expression significantly correlates with poor prognosis for patients. P-values indicate the significance of survival difference between the groups of individuals. In C-H, n indicates the number of samples per group.

We addressed whether expression of the AngioMatrix signature correlates with surrogate markers of blood vessels and angiogenesis in CRC. We determined for each sample the AngioMatrix signature expression level by averaging the expression levels of the 110 genes forming the signature, thereafter referred to as “AngioMatrix expression”, and observed significant correlations with the expression of the EC markers *PECAM1* (Fig. 5A) and *CDH5* (Fig. 5B). We next analyzed the pattern of AngioMatrix expression along CRC formation and progression. This revealed higher expression in normal tissue compared to adenoma and up-regulation during the adenoma-carcinoma transition (Fig. 5C), which was confirmed in an independent cohort (Supplementary Fig. S4A). We observed significantly higher AngioMatrix expression in primary CRC classified as Duke B or Duke C (*versus* A; Supplementary Fig. S4B), and higher expression in advanced primary CRC in an independent cohort (stage 3 or 4 *versus* 0, TNM; Supplementary Fig. S4C). We next asked if AngioMatrix expression could vary according to CRC molecular subtypes [29, 34] and found a significantly higher AngioMatrix expression in the Inflammatory subtype (compared to the Goblet-like or the Transit-amplifying subtypes) and in the Stem-like subtype compared to any other subtype (Fig. 5D). Furthermore, AngioMatrix expression was significantly lower in the C3 and the C1 subtypes and higher in the C4 subtype (Fig. 5E). We then wondered if AngioMatrix expression may vary during the ultimate steps of CRC progression. We found slightly increased AngioMatrix expression in metastatic (compared to non-metastatic) primary CRC (Supplementary Fig. S4D). In CRC metastasis, while no difference is observed in the lung (Supplementary Fig. S4E), AngioMatrix expression is significantly up-regulated in liver metastasis compared to normal tissue (Fig. 5F). The recurrent link between increased AngioMatrix expression and CRC progression prompted us to test a potential correlation with CRC patient survival. We used datasets from two independent cohorts of patients [28, 29], which were stratified using cut-off values into AngioMatrix low or high groups and survival analysis was performed to compare the outcome of these groups. A high expression of the AngioMatrix signature significantly correlated with a shorter relapse-free survival in the two CRC cohorts (Fig. 5, G and H, and Supplementary Figure S4, F and G).

**Figure 6:**
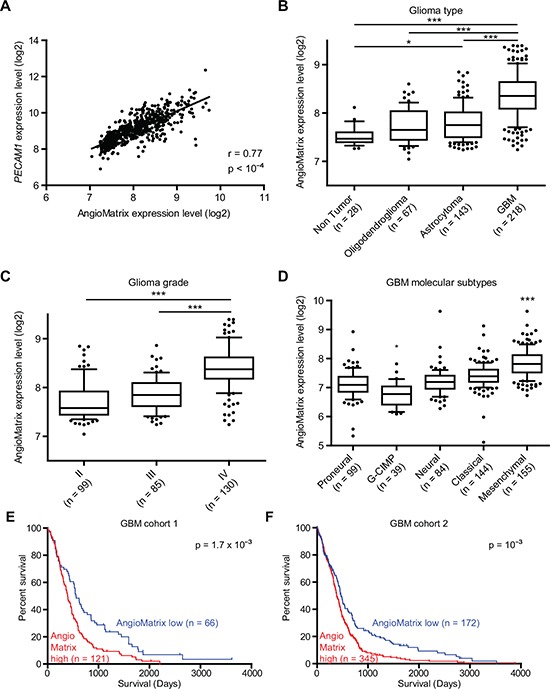
Correlation between AngioMatrix signature expression and EC marker, tumor progression and poor prognosis in human glioma **(A)** correlation between AngioMatrix expression and *PECAM1* in glioma samples. The Pearson correlation coefficient (r) and the p-value are indicated. **(B)** comparison of AngioMatrix expression between non tumor brain samples and glioma histological subtypes. Note the higher levels of AngioMatrix expression in GBM compared to normal brain tissue, oligodendroglioma or astrocytoma. **(C-D)** analysis of AngioMatrix expression according to glioma grade (C) and the different GBM molecular subtypes (D). Note the significantly higher levels in grade IV glioma (C) and in the GBM mesenchymal subtype (D). In B-D, * and *** indicate p-values < 5×10^−2^ and 10^−3^, respectively. **(E-F)** Kaplan-Meier survival analysis of GBM patients. Patients were stratified according to the average expression of the AngioMatrix signature as AngioMatrix high or low using a cutoff value. In each cohort, high AngioMatrix expression significantly correlates with poor prognosis for patients. P-values indicate the significance of survival difference between the groups of individuals. In B-F, n indicates the number of samples per group.

We analyzed AngioMatrix expression in independent glioma datasets and observed again a significant correlation between AngioMatrix expression and the EC markers *PECAM1* (Fig. 6A) and *CDH5* (Supplementary Fig. S5A). Comparing glioma histological subtypes revealed higher AngioMatrix expression in GBM compared to astrocytoma or oligodendroglioma (Fig. 6B), which was confirmed in an independent cohort (Supplementary Fig. S5B). Also, AngioMatrix expression increased with grade (Fig. 6C). Differences in AngioMatrix expression were observed between the GBM molecular subtypes [35], of which the highest expression in the mesenchymal subtype was the most significant (Fig. 6D). The recurrent correlation between AngioMatrix expression and glioma progression led us to test the potential use of the signature to stratify glioma patients and analyze their survival. High AngioMatrix expression significantly correlated with poor prognosis for all glioma patients (Supplementary Fig. S5C) and for subgroups of low-grade glioma: astrocytoma, oligodendroglioma, grade II, grade III or combined grade II and III glioma (Supplementary Fig. S5D-H). Finally, we analyzed GBM from two independent cohorts and found that high AngioMatrix expression significantly correlated with shortened patient survival (Fig. 6, E and F, and Supplementary Figure S5, I and J).

## DISCUSSION

We have used a strategy based on gene expression profiling to comprehensively describe the angiogenic switch in a prototypical murine cancer model [2, 4]. Our microarray analysis first revealed the up-regulation of cell type specific markers in the angiogenic islets, suggesting an expansion of stromal cells. This was confirmed at tissue level using specific markers for endothelial cells, pericytes and macrophages. Of note, no neutrophil marker was retrieved, although neutrophils have been functionally implicated in the RIP1-Tag2 angiogenic switch [7, 8]. Since we have extracted RNA from whole islets for gene expression profiling, we may have missed the low abundant neutrophils (0.4% of RIP1-Tag2 islet cells; ref. [7]). At the molecular level, we noted a recurrent overlap between the AngioSwitch signature and several cellular signaling pathways that have been functionally implicated in RIP1-Tag2 tumor progression, including the PDGF receptor β and its ligand PDGF-BB [36] or endoglin [37]. We also observed and confirmed up-regulation of several genes encoding canonical TGF-β signaling pathway members, suggesting that TGF-β signaling is activated during the RIP1-Tag2 angiogenic switch. This is in line with a previous report showing the up-regulation of *Tgfb1* and the presence of ALK5-positive cells, (expressing TGF-β receptor 1 and therefore susceptible of undergoing canonical TGF-β signaling in the presence of ligand) within RIP1-Tag2 angiogenic islets, and presumably representing stromal cells [21]. We found significant enrichment of fibroblast- and macrophage-specific TBRS, which suggested that these tumor-associated stromal cells may undergo signaling. Accordingly, we demonstrated their presence and that they undergo canonical TGF-β signaling as revealed by the nuclear localization of phosphorylated SMAD3 within these stromal cells in angiogenic but not in non angiogenic RIP1-Tag2 islets. Altogether, these observations strongly support the notion that the AngioSwitch signature is biologically and functionally meaningful, and that the activation of canonical TGF-β signaling within stromal cells may represent a key event driving this transition. Mechanistically it remains to be determined which specific signals trigger TGF-β signaling during the RIP1-Tag2 angiogenic switch and how specific AngioMatrix molecules are implicated. MMP-9 and MMP-2 represent candidate drivers of the transition since both are induced during the RIP1-Tag2 angiogenic switch and MMP9 in particular exerts a crucial role [6] and both MMPs are able to activate latent TGF-β [38]. Furthermore we uncovered that the RIP1-Tag2 angiogenic switch is associated with the up-regulation of genes encoding ECM and ECM-associated molecules. This is in line with our and others findings, as canonical TGF-β signaling regulates the production of ECM and regulators in the microenvironment of tissue under various physio-pathological conditions including cancer [39, 40]. Although beyond the current scope, it will be important to evaluate in the future whether blocking TGF-β signaling potentially impinges on the angiogenic switch affecting the expression of AngioMatrix molecules.

Using an elegant approach combining *in silico* and proteomic analysis, Naba and co-workers defined the matrisome, a comprehensive list of ECM and ECM-associated molecules [23, 24]. Using this resource we assessed the overlap with the AngioSwitch signature to define the AngioMatrix signature and validated the induction of expression for several AngioMatrix proteins during the angiogenic switch, including the ECM glycoproteins fibronectin, tenascin-C, sparc and periostin. Functionally, we demonstrated that *TNC* ablation impairs the RIP1-Tag2 angiogenic switch, in line with our macroscopical characterization of the two islet classes [25]. These data again support the notion that components of the AngioMatrix signature promote the RIP1-Tag2 angiogenic switch.

To evaluate the potential translational relevance of the AngioMatrix signature for cancer patients, we showed that AngioMatrix expression significantly correlated with EC markers in human CRC and glioma, supporting the notion that this signature also correlates with the angiogenesis status within human tumors. During CRC progression, AngioMatrix expression is increased at the adenoma-carcinoma transition, in partial agreement with previous studies showing that the angiogenic switch occurs early along the adenoma-carcinoma sequence [41, 42]. In glioma, AngioMatrix expression is significantly up-regulated in GBM compared to lower grade glioma. This may reflect vascular co-option in low-grade glioma in contrast to angiogenesis that is more important for GBM vascularization [20]. AngioMatrix expression varies according to primary CRC molecular subtypes [29, 34]. Although these studies have followed different approaches to define CRC subtypes, we found significantly higher levels of AngioMatrix expression in the stem-like [34] and the C4 [29] subtypes, the latter being also enriched in stem cell-like signatures [29]. We speculate that higher AngioMatrix expression in stem-like CRC reflects a potential role of some AngioMatrix molecules not only in angiogenesis but also in the regulation of cell fate within (cancer) stem cell niches. Moreover, tenascin-C and periostin are both expressed in the hair follicle stem cell niche in murine skin and are crucial for metastatic breast cancer stem cells colonizing the lung [43–45]. It will be interesting to determine if other AngioMatrix molecules represent normal and cancer stem cell niches components. We found lower AngioMatrix expression levels in the C1 and C3 subtypes, and higher level in the C4 subtype, which correlates with the respective enrichment of the GO sprouting angiogenesis category within these subtypes [29], reinforcing the notion that this signature correlates with angiogenesis in human CRC. Also, higher AngioMatrix expression levels are found in the GBM mesenchymal subtype, described as enriched in EC and angiogenesis markers [35]. Finally, the AngioMatrix signature allows to identify CRC, low-grade glioma and GBM patients with a poorer prognosis. It will be important to determine whether this can be extended to other tumor types and if specific AngioMatrix subsets may improve stratification of patients at higher risk of tumor relapse.

ECM molecules and regulators exert key functions during vascular remodeling in tumors and play instrumental roles in promoting tumor progression by multiple mechanisms as e.g. providing pro-angiogenic niches and favoring tumor cell survival and dissemination. Importantly, ECM molecules represent potential therapeutic targets as functional studies have underlined their importance in the process of blood vessel regrowth after anti-angiogenic therapy [12]. Whether AngioMatrix molecules are potentially relevant in tumor vessel regrowth is unknown and important to be addressed in the future. It is interesting to note that the ECM glycoproteins fibronectin, tenascin-C and periostin, that were found here among the most highly up-regulated genes during the angiogenic switch, have also been identified as crucial for metastatic colonization in other cancer models *in vivo* [44–46]. Further studies are warranted to assess if additional AngioMatrix molecules also contribute to the generation of metastatic niches. Finally, AngioMatrix expression is significantly higher in hepatic metastases, the most common metastatic site for CRC. It will be interesting to determine if some AngioMatrix molecules represent metastasis-specific components as these could represent novel opportunities to develop targeted therapies.

In summary, we have shown that the angiogenic switch, a rate-limiting and early step during PNET progression in a murine model, is associated with a specific transcriptome, which allowed us to define the AngioMatrix signature and show that it correlates with tumor progression and poor prognosis for CRC, low-grade glioma and GBM patients. Our study paves the way for the identification of novel molecular and cellular mechanisms that are key to tumor angiogenesis and might unravel novel opportunities for diagnosis and therapeutic targeting.

## METHODS

A detailed description is available from the Supplementary methods.

### RIP1-Tag2 mice

Experiments involving RIP1-Tag2 animals [3] were done at 8 weeks and in accordance with the guidelines from INSERM (National Institute for Health and Medical Research), as described [25].

### Genome-wide gene expression profiling and data mining

Pools of angiogenic and non angiogenic pancreatic islets were sorted as described [25] and RNA was extracted for labeling and hybridization (Affymetrix arrays). Data are deposited in the Gene Expression Omnibus (NCBI, GSE51637). Significantly deregulated genes were selected using the BRB-ArrayTools software (NCI, USA). The matrisome [23, 24] was used to compare the overlap with the AngioSwitch signature and define the AngioMatrix signature. Enrichments of TBRS from specific stromal cell types [22], the matrisome and its divisions [23] in the profiling dataset of RIP1-Tag2 angiogenic and non angiogenic islets we generated (GSE51637) were analyzed using GSEA [47]. Correlations between AngioMatrix expression and various parameters were analyzed in independent cohorts of CRC [26–30] and glioma [31–33]. Molecular subtypes of CRC [29, 34] and GBM [35] were previously defined. Kaplan-Meier survival analysis was performed by analyzing transcriptomic datasets from independent cohorts of human CRC [28, 29], glioma and subtypes [31] and glioblastoma [31, 33].

### Statistical analysis and graphical representation

Analysis was performed using GraphPad Prism (GraphPad Software, Inc. USA), Epi Info (Centers for Disease Control and Prevention, USA), BRB-ArrayTools (NCI, USA) and GSEA [47]. Histograms represent data expressed as mean +/− SEM. When comparing two groups, data were analyzed using two-tailed Mann Whitney U or unpaired Student t tests. When comparing three groups or more, data were analyzed using one-way ANOVA (with Tukey's post-test) or Kruskal-Wallis (with Dunn's post-test) tests. In box plots, whiskers represent the 10^th^ and 90^th^ centiles, and data points outside this interval are represented. The false discovery rate (FDR) q-value and the log-rank test were used to assess the significance of GSEA enrichments and of survival differences, respectively. P-values and q-values < 0.05 were considered as significant.

## SUPPLEMENTARY METHODS, FIGURES AND TABLE


