# Supplementary material for: AngioMatrix, a signature of the tumor angiogenic switch-specific matrisome, correlates with poor prognosis for glioma and colorectal cancer patients
Source: Oncotarget. 2014 Oct 7;5(21):10529–45. doi: 10.18632/oncotarget.2470 (PMC4279391; doi:10.18632/oncotarget.2470)
Supplement: Supplementary file 1 [file oncotarget-05-10529-s001.pdf]

SUPPLEMENTARY METHODS, FIGURES AND TABLE

RIP1-Tag2 mice

C57BL/6 RIP1-Tag2 mice, obtained from G. Christofori (Basel University, Switzerland), were bred and housed according to standard protocols and were given food and water *ad libidum*. Genotyping was performed by PCR on DNA extracted from mouse tail. The presence of the RIP1-Tag2 transgene was identified using primers “Tag1”: 5′-GGA CAA ACC ACA ACT AGA ATG CAG-3′ and “Tag2”: 5′-CAG AGC AGA ATT GTG GAG TGG-3′. TNC-deficient mice [1] were backcrossed for ten generations into the C57BL/6 mouse strain. These mice were bred with wild-type TNC expressing (TNC+/+) mice or mice lacking (TNC-/-). The presence or deletion of TNC was determined using primers “TNCKO_TNCup”: 5′-CTG CCA GGC ATC TTT CTA GC-3′, “TNCKO_TNCdown”: 5′-TTC TGC AGG TTG GAG GCA AC-3′ and “TNCKO_TNCNeoPA”: 5′-CTG CTC TTT ACT GAA GGC TC-3′.

Isolation of pancreatic islets

Langerhans islets were isolated from 8 (7.9–8.4) week-old RIP1-Tag2 mice using Liberase (RI or TL, Roche), dissolved in DMEM (1 g/l glucose) and diluted to 0.82–1.0 Wünsch units/ml. Mice were sacrificed by cervical dislocation and pancreata were perfused via the bile duct with 2 ml Liberase solution, removed and digested at 37°C for 17–24 min. Digestion was stopped by addition of DMEM/15% FCS and strong shaking. The digested tissue was washed with DMEM, filtered through a mesh (with 380 µm pores), mixed with 10 ml Histopaque 1077 (Sigma) and covered with 10 ml of DMEM to create a gradient. Islets were separated by centrifugation (30 minutes, 1500 g at room temperature), recovered from the gradient interphase, washed with DMEM and transferred into islet culture medium (RPMI 1640 containing 11.1 mM glucose, 15% FCS, 1% penicillin/streptomycin, 7.5% NaHCO3, 66 µM β-mercaptoethanol). Intact islets were observed under a stereomicroscope (Leica), classified into non-angiogenic (completely white appearance) or angiogenic (few reddish spots up to completely reddish) and, were hand-picked and isolated. For each mouse, non-angiogenic and angiogenic islet pools were collected separately in sterile microcentrifuge tubes. Samples were shortly centrifuged, medium removed, washed with PBS, snap frozen in liquid nitrogen and kept at -80°C.

Gene expression profiling of isolated RIP1-Tag2 islets

Pools of non-angiogenic and of angiogenic islets isolated from 8 week-old RIP1-Tag2 mice were prepared and total RNA was extracted (NucleoSpin RNA XS kit, Macherey-Nagel, Düren, Germany). Isolated islets from 1 to 4 mice were pooled to obtain 63 to 211 non-angiogenic islets or 23 to 93 angiogenic islets for each sample profiled on a microarray. Quality of extracted RNA was assessed using the Agilent 2100 Bioanalyzer (RNA 6000 Nano Kit). All microarray experiments were performed by the IGBMC Microarray facility (Illkirch, France) following manufacturer (Affymetrix) instructions. Briefly, for each sample, 200 ng RNA was used to prepare labeled cRNA probes hybridized to Mo-Gene 1.0 ST arrays. For each experiment, three biological replicates (3 pools of non-angiogenic islets and 3 pools of angiogenic islets) were profiled. The experiment (from islets isolation to RNA profiling using microarrays) was repeated twice independently, giving rise to 6 microarrays for each condition (NA or A) in total, that were normalized and analyzed together. Raw data were normalized by the RMA method using the Expression Console software (Affymetrix, build 1.2.1.20). Data are deposited in the NCBI Gene Expression Omnibus repository (GSE51637). The BRB-ArrayTools software (NCI, USA) was used to select significantly deregulated genes (ratio angiogenic/non-angiogenic > 1.4 - fold, p-value < 0.05). The Molecular Signature database [2] was used to analyze the Gene Ontologies significantly enriched in the AngioSwitch signature. The matrisome [3,4], a list of genes known and inferred to encode ECM molecules was used to compute overlaps with the AngioSwitch signature, in order to generate the AngioMatrix signature (110 murine genes induced during the RIP1-Tag2 angiogenic switch and belonging to the matrisome division). GSEA [2,5] (version 2.0.13) was used to analyze enrichment of the matrisome and its divisions in the angiogenic versus non-angiogenic islets microarray dataset we generated. Mapping of TGF- β signaling associated genes in the AngioSwitch signature was performed using a custom-built map in GenMAPP 2 [6].

RT-qPCR analysis

Expression validation of genes found deregulated from the microarray analysis were done on 3 islets pools that were used for the microarray profiling together with 3 islets pools that were independently prepared, comparing in total 6 pools of non-angiogenic islets to 6 pools of angiogenic islets. Reverse Transcription reactions were performed on 200 ng of RNA using MultiScribe reverse transcriptase (Applied Biosystems) and following the manufacturer's instructions. Primer sequences (Table S1) were designed using Roche Probefinder (v2.45 or later). Primer pairs were initially tested and validated for specificity and efficiency using cDNA dilutions prepared from RIP1-Tag2 tumor derived RNA. Quantitative PCR were performed using a 7500 Real Time PCR System (Applied Biosystems) using SYBR green reagent (Applied Biosystems) and results analyzed using the $2^{\Delta\Delta Ct}$ method [7]. The *Rpl19* gene was used as reference gene as it was found to be the gene with the most stable expression (compared to the other reference genes tested, *Hmbs* and *Tbp*) in RIP1-Tag2 pools of angiogenic and non angiogenic islets. Relative expression levels ($2^{-\Delta\Delta ct}$) were calculated for each individual sample, and compared between non-angiogenic and angiogenic islet pools. All RT-qPCR experiments were performed twice independently and measures subsequently were averaged.

Tissue analysis

Pancreata were fixed for 2h at room temperature in 4% paraformaldehyde in PBS 1X, immersed in 20% sucrose for 12h at 4°C and embedded in Tissue-Tek OCT (Sakura Fine Tek). Alternatively, pancreata were fixed for 2h at 4°C in 4% paraformaldehyde in PBS 1X, dehydrated and embedded in paraffin. Tissue was cut and sections were routinely stained with hematoxylin and eosin (H&E), or used for immuno-staining analysis. Quantification of non-angiogenic and angiogenic islets was performed using a histological analysis of H&E stained tissue sections from paraffin embedded pancreata, comparing tissue from 5 TNC+/+ and 8 RIP1-Tag2 TNC-/- mice. Islets were considered as angiogenic when their biggest diameter was above 350 µm. This cutoff was chosen as it was enabling to correctly classify all angiogenic islets and with a minimal number of false positive (non angiogenic islets misclassified as angiogenic; < 2%) from a setup analysis comparing a previously described set of criteria [8] to the measure of islet biggest diameter in a series of tissue sections from five 8-week old RIP1-Tag2 mice. For immunostainings, primary antibodies used were: rat monoclonal anti-CD31 (BD Pharmingen 550274, 1/50), rabbit monoclonal anti-vimentin (Epitomics 2707-1, 1/500), rabbit polyclonal anti-NG2 (Millipore AB5320, 1/200), rat monoclonal anti F4/80 (AbD serotec MCA497G, 1/200), Cy3-conjugated monoclonal anti-α-Smooth Muscle Actin (αSMA, Sigma C6198, 1/400), rabbit polyclonal anti-phospho-S423/S425 SMAD3 (Rockland, 600-401-919, 1/100), rabbit polyclonal anti-Fibronectin (Sigma F3648, 1/200), goat polyclonal anti-SPARC (R&D systems, 1/200), rat monoclonal anti-Tenascin-C [9] (MTn 12, purified from hybridoma culture supernatants; 20µg/mL), rabbit polyclonal anti-Collagen IV 2a [10] (1/200), rabbit polyclonal anti-Laminin-α4 [11] (1/500), mouse monoclonal anti-Periostin [12] used for IHC (1/500). For immunofluorescent detection primary antibodies were incubated overnight at 4°C, Cy3-conjugated anti-αSMA and secondary antibodies (Interchim Dylight488-anti-rabbit, Cy3-anti-rat, Cy3-anti-goat, Cy5-anti-rabbit 1/2000) were incubated for 1h at room temperature and cell nuclei were stained with DAPI. Immunohistochemistry for detection of periostin was performed on paraffin-embedded sections using Vectastain developing system (Vector Laboratories) followed by tissue staining with hematoxylin. For analysis of nuclear localization of phosphorylated SMAD3, Z-series acquisitions (seven Z-plans with a step of 0.34µm) were performed with an Axio Imager.Z2 microscope (Zeiss) equipped with 40x objective and an ApoTome module. Pictures presented correspond to one Z-section with nuclear focus in αSMA or F4/80 positive cells.

Analysis of AngioMatrix signature expression in publicly-available gene expression datasets of human samples, stratification of patients and survival analysis

The murine AngioMatrix signature was first converted to human homologs using the Homologene database (release 67; NCBI, USA). AngioMatrix expression level was calculated by averaging the expression level of the 110 genes forming the signature in a given sample.

To analyze AngioMatrix expression along CRC progression a first dataset comprising colorectal adenoma, primary CRC of different Duke stage, liver and lung metastases, and corresponding normal tissue samples was used [13]. In addition, independent datasets comprising normal, adenoma and primary CRC [14], and metastatic *versus* non metastatic primary CRC [15] were also analyzed. Correlation between AngioMatrix expression levels and *PECAM1* or *CDH5* expression was determined in normal intestinal mucosa, adenoma and primary CRC samples [13]. The primary CRC cohort 1 [16] was used to analyze AngioMatrix expression levels in the five

different CRC molecular subtypes identified and defined by Sadanandam et al. [17]. The primary CRC cohort 2 [18] was used to analyze AngioMatrix expression levels in the six different CRC molecular subtypes identified and defined by these authors [18].

Correlations between AngioMatrix expression level and *PECAM1* or *CDH5* expression, and AngioMatrix expression levels in different glioma subtypes were analyzed in the glioma cohort 1 [19]. In addition, AngioMatrix expression level in different glioma histologic types was determined in an independent cohort [20]. Analysis of AngioMatrix expression level in the different GBM molecular subtypes [21] was performed using the GBM cohort 2 [22].

Kaplan-Meier analysis of cancer patient survival was performed as previously described [23], using Epi Info (version 3.5.4; Centers for Disease Control and Prevention, USA) and GraphPad (GraphPad Software, Inc. USA) to analyze genome-wide gene expression datasets from human colorectal cancers (cohort 1, ref. [16]; cohort 2, ref. [18]), all glioma or glioma subgroups [19] and glioblastoma (cohort 1, ref. [19]; cohort 2, ref. [22]). For each cohort, a cutoff was used to assign a tumor/patient to the AngioMatrix high group if the average expression of the 110 genes defining the human AngioMatrix signature was above the cutoff, and conversely to the AngioMatrix low group if this value was below the cutoff. The cutoff values were either empirically determined as to provide the best possible stratification between AngioMatrix high and low groups for each cohort (Figure 5 and 6) or the median as a cutoff based on data distribution in the cohorts (Supplementary Figure 4 and 5). Note that both stratification methods gave similar results: in either case a poorer prognosis was observed for the AngioMatrix high patient group. The log-rank test was used to assess the significance of survival differences between patient groups.

REFERENCES

1. Forsberg E, Hirsch E, Fröhlich L, Meyer M, Ekblom P, Aszodi A, Werner S, Fässler R. Skin wounds and severed nerves heal normally in mice lacking tenascin-C. Proc Natl Acad Sci U S A. 1996; 93:6594–6599.
2. Subramanian A, Tamayo P, Mootha VK, Mukherjee S, Ebert BL, Gillette MA, Paulovich A, Pomeroy SL, Golub TR, Lander ES, Mesirov JP. Gene set enrichment analysis: a knowledge-based approach for interpreting genome-wide expression profiles. Proc Natl Acad Sci U S A. 2005; 102:15545–15550.
3. Hynes RO,, Naba A. Overview of the matrisome—an inventory of extracellular matrix constituents and functions. Cold Spring Harb Perspect Biol. 2012; 4:a004903.
4. Naba A, Clauser KR, Hoersch S, Liu H, Carr SA, Hynes RO. The matrisome: in silico definition and in vivo characterization by proteomics of normal and tumor extracellular matrices. Mol Cell Proteomics. 2012; 11 M111.014647.
5. Mootha VK, Lindgren CM, Eriksson K-F, Subramanian A, Sihag S, Lehar J, Puigserver P, Carlsson E, Ridderstråle M, Laurila E, Houstis N, Daly MJ, Patterson N, et al. PGC-1alpha-responsive genes involved in oxidative phosphorylation are coordinately downregulated in human diabetes. Nat Genet. 2003; 34:267–273.
6. Dahlquist KD, Salomonis N, Vranizan K, Lawlor SC, Conklin BR. GenMAPP, a new tool for viewing and analyzing microarray data on biological pathways. Nat Genet. 2002; 31:19–20.
7. Pfaffl MW. A new mathematical model for relative quantification in real-time RT-PCR. Nucleic Acids Res. 2001; 29:e45.
8. Lopez T, Hanahan D. Elevated levels of IGF-1 receptor convey invasive and metastatic capability in a mouse model of pancreatic islet tumorigenesis. Cancer Cell. 2002; 1: 339–353.
9. Aufderheide E, Ekblom P. Tenascin during gut development: appearance in the mesenchyme, shift in molecular forms, and dependence on epithelial-mesenchymal interactions. J Cell Biol. 1988; 107:2341–2349.
10. De Arcangelis A, Neuville P, Boukamel R, Lefebvre O, Kedinger M, Simon-Assmann P. Inhibition of laminin alpha 1-chain expression leads to alteration of basement membrane assembly and cell differentiation. J Cell Biol. 1996; 133:417–430.
11. Sixt M, Engelhardt B, Pausch F, Hallmann R, Wendler O, Sorokin LM. Endothelial cell laminin isoforms, laminins 8 and 10, play decisive roles in T cell recruitment across the blood-brain barrier in experimental autoimmune encephalomyelitis. J Cell Biol. 2001; 153:933–946.
12. Malanchi I, Santamaria-Martínez A, Susanto E, Peng H, Lehr H-A, Delaloye J-F, Huelsken J. Interactions between cancer stem cells and their niche govern metastatic colonization. Nature. 2012; 481:85–89.
13. Sheffer M, Bacolod MD, Zuk O, Giardina SF, Pincas H, Barany F, Paty PB, Gerald WL, Notterman DA, Domany E. Association of survival and disease progression with chromosomal instability: a genomic exploration of colorectal cancer. Proc Natl Acad Sci U S A. 2009; 106:7131–7136.
14. Galamb O, Wichmann B, Sipos F, Spisák S, Krenács T, Tóth K, Leiszter K, Kalmár A, Tulassay Z, Molnár B. Dysplasia-carcinoma transition specific transcripts in colonic biopsy samples. PloS One. 2012; 7:e48547.
15. Watanabe T, Kobunai T, Yamamoto Y, Matsuda K, Ishihara S, Nozawa K, Iinuma H, Konishi T, Horie H,

Ikeuchi H, Eshima K, Muto T. Gene expression signature and response to the use of leucovorin, fluorouracil and oxaliplatin in colorectal cancer patients. Clin Transl Oncol Off Publ Fed Span Oncol Soc Natl Cancer Inst Mex. 2011; 13:419–425.

16. Jorissen RN, Gibbs P, Christie M, Prakash S, Lipton L, Desai J, Kerr D, Aaltonen LA, Arango D, Kruhøffer M, Orntoft TF, Andersen CL, Gruidl M et al. Metastasis-Associated Gene Expression Changes Predict Poor Outcomes in Patients with Dukes Stage B and C Colorectal Cancer. Clin Cancer Res Off J Am Assoc Cancer Res. 2009; 15:7642–7651.
17. Sadanandam A, Lyssiotis CA, Homicsko K, Collisson EA, Gibb WJ, Wullschleger S, Ostos LCG, Lannon WA, Grotzinger C, Del Rio M, Lhermitte B, Olshen AB, Wiedenmann B, et al. A colorectal cancer classification system that associates cellular phenotype and responses to therapy. Nat Med. 2013; 19:619–625.
18. Marisa L, de Reyniès A, Duval A, Selves J, Gaub MP, Vescovo L, Etienne-Grimaldi M-C, Schiappa R, Guenot D, Ayadi M, Kirzin S, Chazal M, Fléjou J-F, et al. Gene expression classification of colon cancer into molecular subtypes: characterization, validation, and prognostic value. PLoS Med. 2013; 10:e1001453.
19. Madhavan S, Zenklusen J-C, Kotliarov Y, Sahni H, Fine HA, Buetow K. Rembrandt: helping personalized medicine become a reality through integrative translational research. Mol Cancer Res. 2009; 7:157–167.
20. Bredel M, Bredel C, Juric D, Harsh GR, Vogel H, Recht LD, Sikic BI. Functional network analysis reveals extended gliomagenesis pathway maps and three novel MYC-interacting genes in human gliomas. Cancer Res. 2005; 65:8679–8689.
21. Brennan CW, Verhaak RGW, McKenna A, Campos B, Noushmehr H, Salama SR, Zheng S, Chakravarty D, Sanborn JZ, Berman SH, Beroukhim R, Bernard B, Wu C-J, et al. The somatic genomic landscape of glioblastoma. Cell. 2013; 155:462–477.
22. Cancer Genome Atlas Research Network: Comprehensive genomic characterization defines human glioblastoma genes and core pathways. Nature. 2008; 455:1061–1068.
23. Hussenet T, Dembélé D, Martinet N, Vignaud J-M, du Manoir S. An adult tissue-specific stem cell molecular phenotype is activated in epithelial cancer stem cells and correlated to patient outcome. Cell Cycle Georget Tex. 2010; 9:321–327.

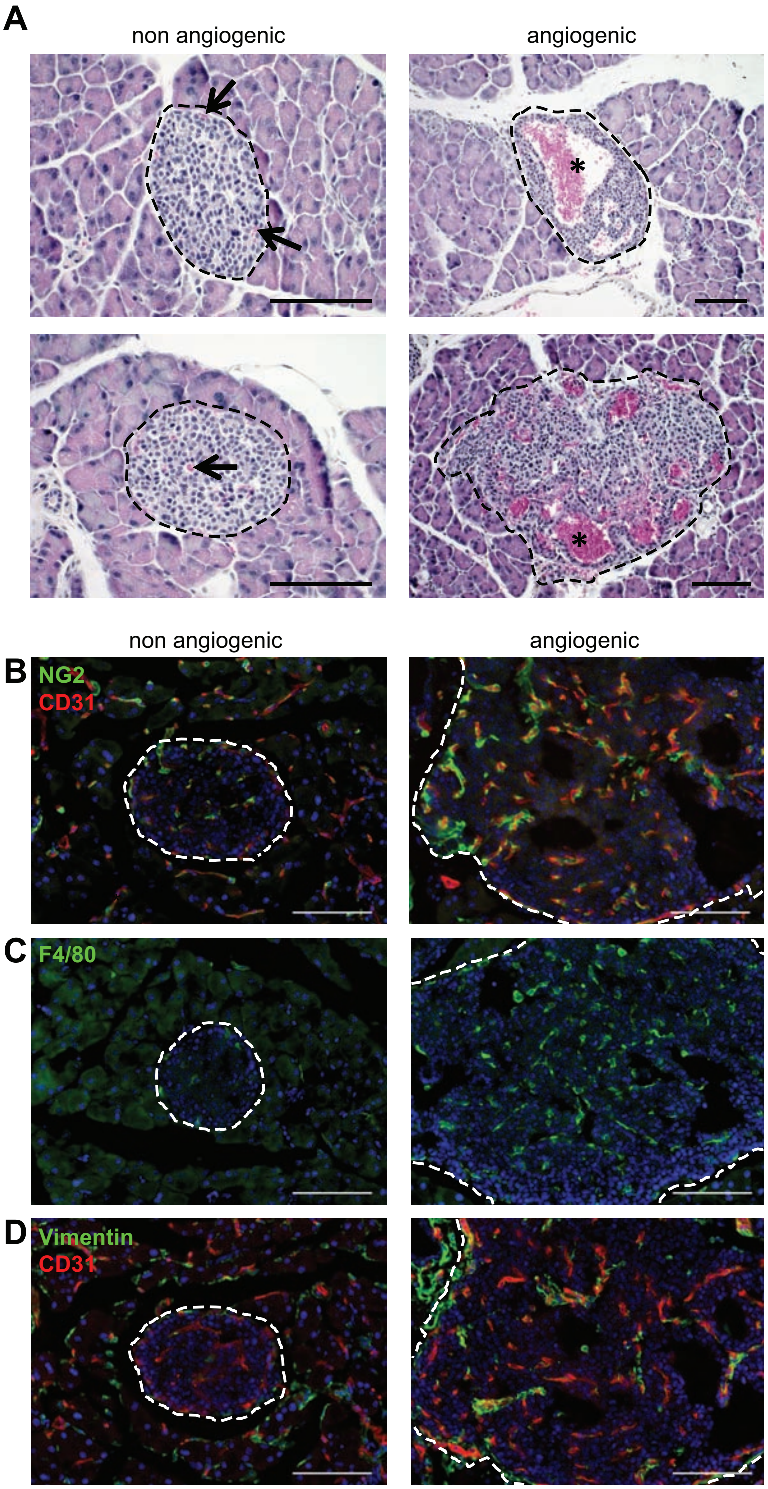

Supplementary Figure S1: Histological patterns of RIP1-Tag2 angiogenesis and increased expression of stromal cell markers during the angiogenic switch. **(A)** patterns of non-angiogenic (left) and angiogenic (right) islets in H&E stained pancreata tissue sections from 8 week old RIP1-Tag2 mice. Examples of normal capillaries in non angiogenic islets (arrows) and of hemorrhaging/blood lakes (asterisks) in angiogenic islets are highlighted. **(B-D)** immunofluorescence analysis of stromal cell markers in non angiogenic and angiogenic RIP1-Tag2 islets. Representative composite images obtained after CD31 (EC) and NG2 (pericyte) co-staining (D), F4/80 (macrophages) (E), and vimentin (perivascular smooth muscle cells) and CD31 (EC) (F) are shown. Nuclei were counterstained with Dapi (blue). RIP1-Tag2 pancreatic islets are encircled by dashed lines. Scale bars, 100 µm.

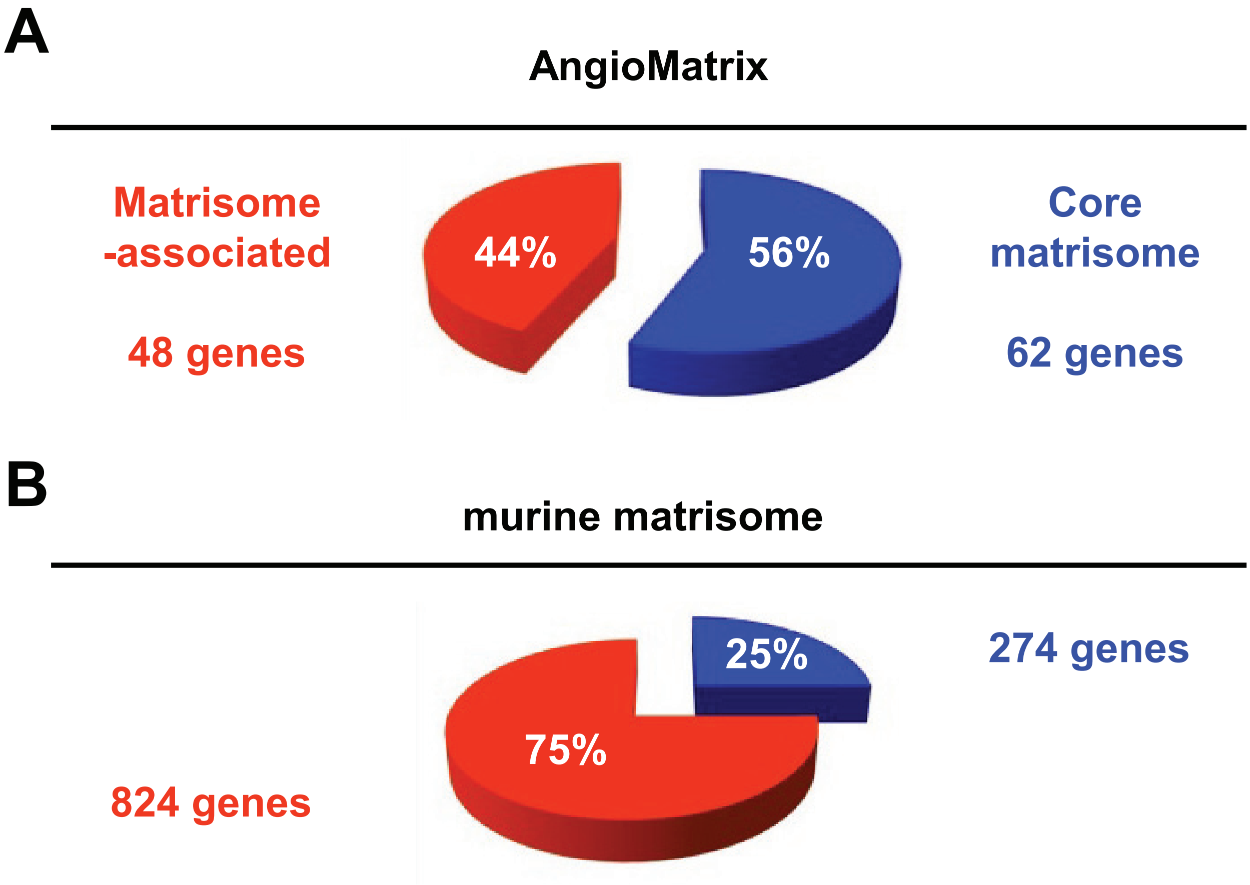

Supplementary Figure S2: Composition of the AngioMatrix signature according to divisions of the matrisome. (A) a majority of genes forming the AngioMatrix signature belong to the core matrisome division (56%, 62 genes), and a minority to the matrisome-associated division (44%, 48 genes). **(B)** the relative proportion of core matrisome (25%, 274 genes) and matrisome-associated (75%, 824 genes) components in the entire murine matrisome are shown for comparison.

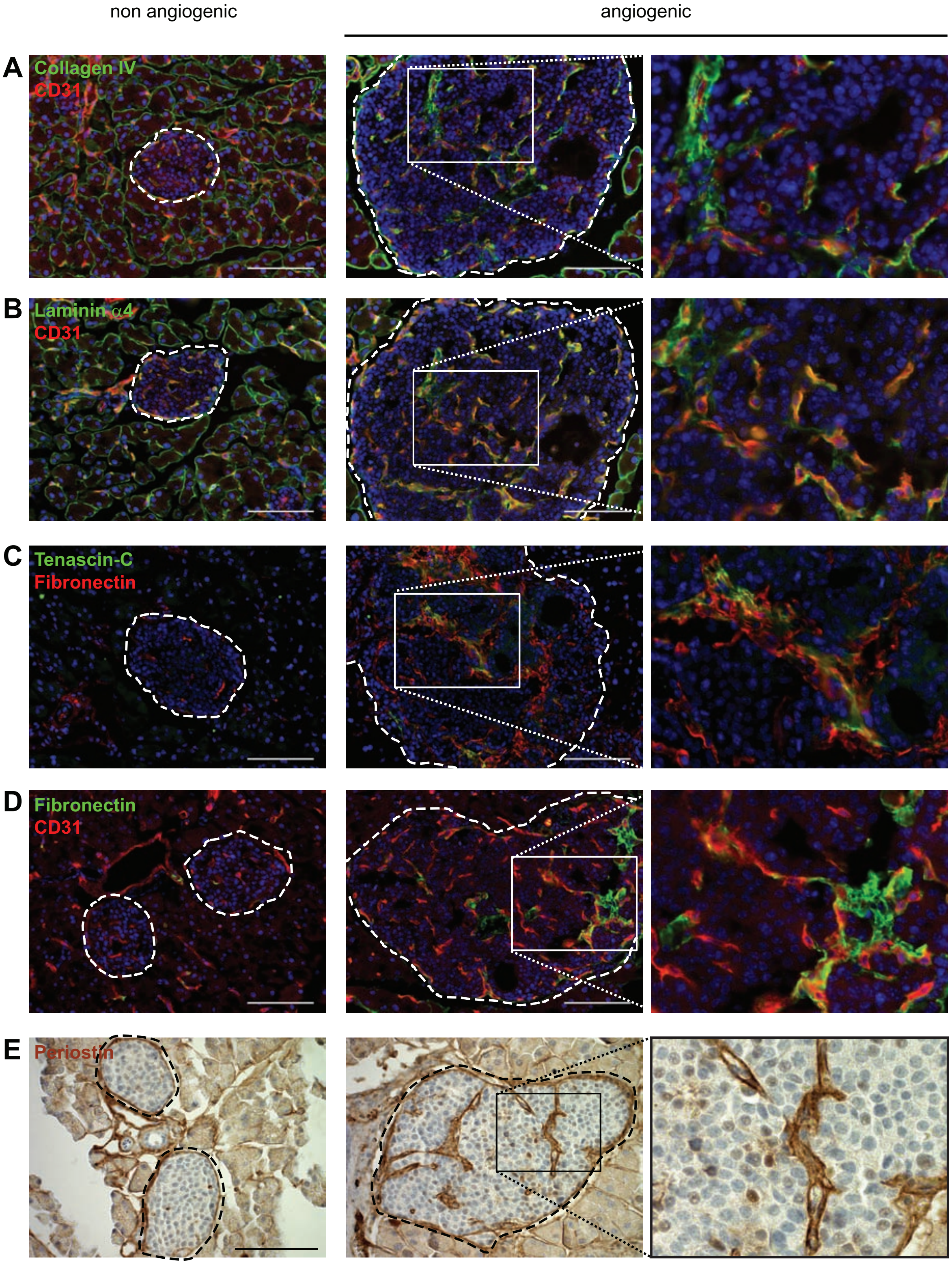

Supplementary Figure S3: Analysis of AngioMatrix protein expression patterns in the RIP1-Tag2 angiogenic switch. (A-B) Expression pattern of the vascular basement membrane molecules Collagen IV (A) and Laminin α4 (B) together with the EC marker CD31 in non angiogenic and angiogenic islets. **(C-E)** Expression pattern of the ECM glycoproteins fibronectin and tenascin-C (C), fibronectin and CD31 (D) and periostin (E) in non angiogenic and angiogenic islets. RIP1-Tag2 pancreatic islets are encircled by dashed lines, and the right column present higher magnification pictures of the corresponding boxed areas in the pictures from angiogenic islets (middle column). Scale bars, 100 μm.

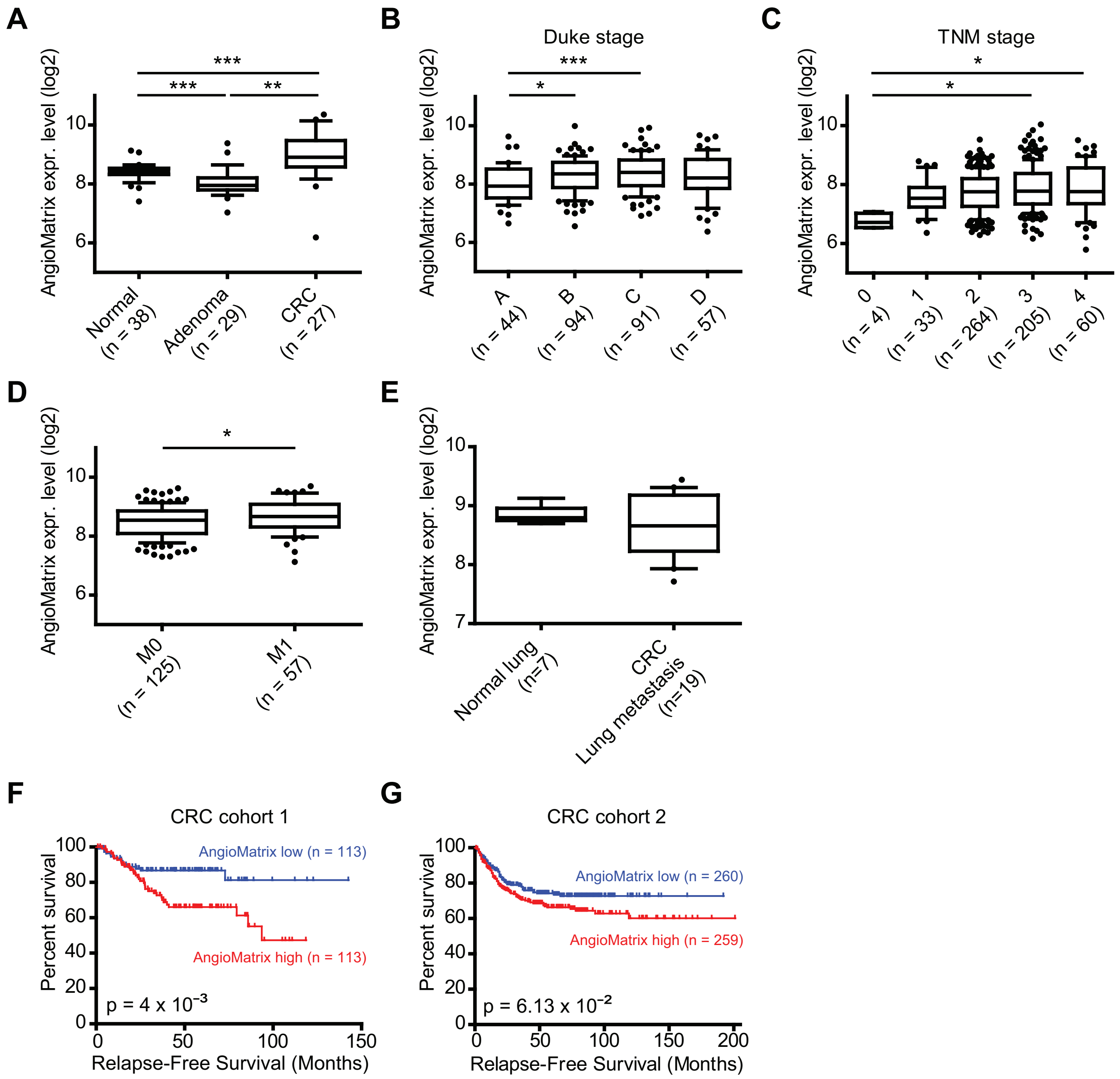

Supplementary Figure S4: Analysis of AngioMatrix expression levels along CRC progression and correlation to clinical parameters. **(A)** AngioMatrix expression level along primary CRC establishment: comparison of normal colonic samples, colorectal adenoma and primary CRC. AngioMatrix expression levels are decreased in adenoma compared to normal samples and increased in primary CRC. *** and ** denote p-values < 10^{-3} and 10^{-2}, respectively, Kruskal-Wallis test with Dunn's post test. **(B-C)** AngioMatrix expression level according to primary CRC stage (B: CRC cohort 1, Duke classification; C: CRC cohort 2, TNM classification). Note the significantly higher levels of AngioMatrix expression in Duke B or C compared to Duke A tumors (B) and in TNM stage 3 or 4 compared to stage 0 (CIS) tumors. *** and * denote p-values < 10^{-3} and 5.10^{-2}, respectively, Kruskal-Wallis test with Dunn's post test. **(D)** comparison of AngioMatrix expression level between non metastatic (M0) and metastatic (M1) primary CRC. A sligt increase of AngioMatrix expression level is observed for metastatic primary CRC. * indicates $p < 0.05$, unpaired Student t-test. **(E)** comparison of AngioMatrix expression level between normal lung and CRC lung metastasis samples. No significant difference is observed. **(F-G)** Kaplan-Meier survival analysis upon stratification of human CRC patients from two independent cohorts using the median expression of the AngioMatrix signature as a cut-off. A significant stratification is observed in the first cohort (F) and a trend in the second cohort (G). Numbers between brackets indicate the number of patients in each group. P-values were calculated with the log-rank test to assess the significance of the observed survival differences between the groups.

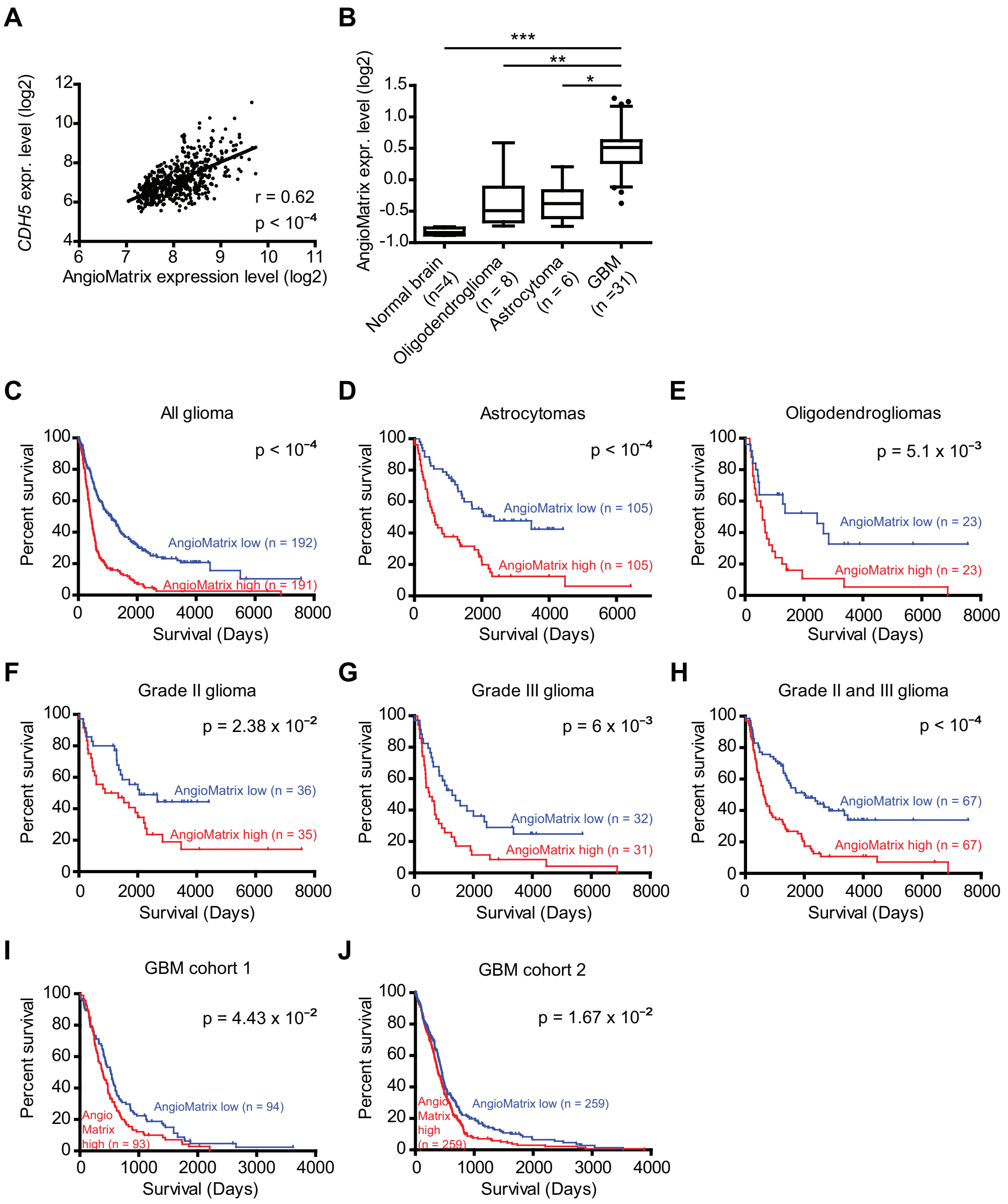

Supplementary Figure S5: Analysis of AngioMatrix expression levels in human glioma and correlation to clinical parameters. **(A)** correlation between AngioMatrix and *CDH5* (encoding vascular endothelial-cadherin) expression levels in glioma. The value of the pearson correlation coefficient (r) and the p-value are indicated. **(B)** AngioMatrix expression level according to glioma histological subtype. Note the significantly higher levels of AngioMatrix expression in GBM compared to any other type. ***, ** and * denote p-values < 10^{-3}, 10^{-2} and 5.10^{-2}, respectively, Kruskal-Wallis test with Dunn's post test. **(C-J)** Kaplan-Meier survival analysis upon stratification of human glioma patients from two independent cohorts using the median expression of the AngioMatrix signature as a cut-off. The glioma cohort 1, composed of different glioma histological types and both lower and higher grade glioma was exhaustively analyzed: glioma patients were stratified by analyzing all glioma samples together (C), or according to subtypes (D-I), defined at histological level (D, E and I) or according to grade for low grade glioma (F-H). In each case, high AngioMatrix expression significantly correlates with poor prognosis for glioma patients. The cohort 2 is composed of high grade glioma (GBM) only (J). Note that for GBM, a significant stratification is observed in both the first (I) and the second (J) cohorts. Numbers between brackets indicate the number of patients in each group. P-values were calculated with the log-rank test to assess the significance of the observed survival differences between the groups.

Supplementary Table S1. Sequences of primer pairs used for qPCR analyses.

Gene	Forward primer (5′ – 3′)	Reverse primer (5′ – 3′)
Ccl2	ggctggagagctacaagagg	ctcttgagcttggtgacaaaaa
Col8a1	gccagccaagcctaaatgt	tgatgaacagtattcccagca
Dlk1	cgggaaattctgcgaaatag	tgtgcaggagcattcgtact
Fn1	gatgccgatcagaagtttgg	ggttgtgcagatctcctcgt
Frzb	caccgtcaatctttataccacct	tcagctatagagccttctaccaaga
Lox	tactcctgggagtggcaca	gacgtgtcctccagacagaag
Ogn	aggaattaaagcaaacacattcaa	tttctggtaaattaggaggcaca
Pdgfb	cggcctgtgactagaagtcc	gagcttgaggcgtcttgg
Pdgfrb	tcaagctgcaggtcaatgtc	ccattggcagggtgactc
Plat	gctacggcaagcatgagg	ggacgggtacagtctgacg
Postn	aatgctgccctggctatatg	gtatgaccctttttccttcaa
Rpl19	accctggcccgacgg	taccttcctcttccctatgcc
Serpine1	ggcacctttgaatactcagga	tttcccagagaccagaacca
Serpinf1	cagagtgcaggctgtgagag	ggctccagtccagaggagtag
Sfrp1	acgagttgaagtcagaggccatc	acagtcggcaccgttcttcag
Sfrp5	gatctgtgcccagtgtgaga	ttaatgcgcatcttgaccac
Tek	gtatggactctttagccggctt	ttcgcccattctctggtcac
Tnc	gcgcagacacacaccctagc	tttccaggtcgggaaaagca
Tgfb1	tgacgtcactggagttgtacgg	ggttcatgtcatggatggtgc
Tgfb2	tcctacagactggagtcacaaca	gcagcaattatcctgcacatt
Tgfb3	gcagacacaacccatagcac	gggttctgcccacatagtaca
Tgfbi	aggaagatctgcggcaagt	tctctcctgggaccttttcat
Thbs4	cagacaactgcaggctcgt	gatatctcctaccccgtcattg
Timp1	gcaaagagctttctcaaagacc	agggatagataaacagggaaacact
Vcam1	ggaagctggaacgaagtatcc	tccagcctgtaaactgggtaa
Vim	ccaacctttttcttccctgaac	ttgagtgggtgtcaaccaga
